# Periodontal biomechanics: finite element simulations of closing stroke and power stroke in equine cheek teeth

**DOI:** 10.1186/1746-6148-8-60

**Published:** 2012-05-20

**Authors:** Vanessa Cordes, Matthias Lüpke, Moritz Gardemin, Hermann Seifert, Carsten Staszyk

**Affiliations:** 1 Institute of Anatomy, University of Veterinary Medicine Hannover, Bischofsholer Damm 15, Hannover, D-30173, Germany; 2 Institute for General Radiology and Medical Physics, University of Veterinary Medicine Hannover, Bischofsholer Damm 15, Hannover, D-30173, Germany; 3 Department of Veterinary-Anatomy, -Histology and -Embryology, Faculty for Veterinary Medicine, Justus-Liebig-University Giessen, Frankfurter Str. 98, Hannover, Giessen, D-35392, Germany

**Keywords:** Finite element analysis, Horse, Periodontal ligament, Tooth, Chewing cycle, Periodontal disease, Periapical infection

## Abstract

**Background:**

In equine dentistry periodontal diseases, especially periapical inflammation, are frequently occurring problems. Anachoresis is believed to be the most common cause for the development of such disorders. Nevertheless, there is still no substantiated explanation why settlement of pathogen microorganisms occurs in equine periodontal tissues. It is expected that excessive strains and stresses occurring in the periodontal ligament (PDL) during the horse’s chewing cycle might be a predisposing factor. In this study this assumption was examined by finite element (FE) analyses on virtual 3-D models of equine maxillary and mandibular cheek teeth, established on the basis of μCT datasets. Calculations were conducted both under conditions of closing and power stroke.

**Results:**

Results showed a uniform distribution of low stresses and strain energy density (SED) during closing stroke, whereas during power stroke an occurrence of high stresses and SED could be observed in the PDL near the alveolar crest and in periapical regions.

**Conclusion:**

The concentration of forces during power stroke in these specific areas of the PDL may cause local tissue necrosis and inflammation and thus establish a suitable environment for the settlement of microorganisms.

## Background

Dentition plays a very important role in equine digestion. Horses do not have a forestomach system like ruminants, which enables a microbiological breakdown of plant cell walls before reaching the resorptive small intestine [1]. Thus, cheek teeth are the only tools for releasing nutritive cell contents by grinding the herbivore diet [1]. An effective disruption of forage is therefore necessary for a sufficient energy supply [2].

One of the main problems in equine oral health are painful periodontal diseases [3-5], hampering normal masticatory action [6]. Especially in geriatric horses the occurrence of periodontal disorders is exceptionally high with described prevalence of about 60% [6].

Periodontal diseases can be subdivided into those proceeding from the gingival sulcus into the periodontal space and those occurring exclusively in the periapical region.

Recent studies have shown that periapical infections can be caused either by periodontal spread, infundibular caries or occlusal fissure fractures [7-9]. Nonetheless, remarkably the aetiology of most cases of the periapical infection (68%) remained unexplained [7]. For these cases anachoresis is suggested to be causative [7-9]. Anachoretic infections depend on a suitable environment for microbiological settlement in terms of necrotic tissue areas [10].

We propose that excessive strains and stresses occurring in the periodontal ligament (PDL) during the horse’s chewing cycle could cause pathological tissue changes and thus be a predisposing factor for equine periodontal diseases.

In this study, computer-based finite element (FE) calculations of stresses and strain energy densities (SED) occurring in the PDL of different-aged teeth during load bearing phases of the equine masticatory cycle (closing stroke and power stroke) were conducted. The results will hopefully give useful contributions for understanding the etiology of periodontal diseases. These calculations were generated on the basis of our previously established finite element models of equine maxillary and mandibular cheek teeth [11,12].

## Methods

### 3-D models

The establishment of virtual 3-D models of different aged mandibular and maxillary equine cheek teeth (tooth age group A: 0–5 years; age group B: 6–15 years; age group C: > 15 years), suitable for FE-simulations, was conducted on the basis of μCT-datasets [12]. In all 3-D models the tooth was assumed to consist of one homogenous material, while when constructing the jaw bone a separation into cortical alveolus (lamina dura), cancellous bone and compact bone was made. The PDL, connecting the tooth with the surrounding cortical alveolus, was built uniformly around the tooth except for periodontal parts around the apical region of the tooth and under the root-bi-/trifurcation, which were segmented as different, modified materials [11]. Surface and volume meshes, necessary for finite element analyses, were generated out of the 3-D models as described by Lüpke et al. (2010) [12]. The quality of all meshes was tested afterwards by a quality measuring function of the computer program COMSOL Multiphysics (version 3.4, COMSOL AB, Stockholm, Sweden) to ensure that mesh topology did not negatively affect the FE solutions.

### Finite element analyses

Finite element simulations of the horse’s chewing cycle were performed for three defined age groups (A-C) using the computer program COMSOL Multiphysics (version 3.4, COMSOL AB, Stockholm, Sweden).

For calculating stresses and SED defined values of Young’s modulus and Poisson’s ratio (values describing the elastic behavior of materials) had to be allocated to each structure in the 3-D meshes (Table 1). Young’s moduli and Poisson’s ratios for the tooth [13-15] and for the bony structures [16,17] as well as the Poisson’s ratio for the PDL [18,19] were shown to be uniform in several studies and were therefore taken from literature. In this study both the tooth and all bony structures were assumed to have linear elastic material properties.

**Table 1:** Values of Poisson’s ratio and Young’s modulus used in FE calculations

	Poisson’s ratio	Young’s modulus (MPa)	References
Tooth	0.3	20000	[13-15]
Compact bone	0.3	20000	[16,17]
Cancelous bone	0.3	1000	[16,17]
PDL	0.45	3.1 (age group A)	[11]
		2.9 (age group B)	
		2.6 (age group C)	

The published values for Young’s modulus of the PDL vary in a wide range dependent on species and measuring methods [20-22]. Thus this value was specifically determined by intrusive movement experiments for each of the used models as described elsewhere [11]. Due to the viscoelastic properties of the PDL [23-25] a non-linear elastic behavior was proven. Nevertheless, for later FE calculations, especially regarding the quality but not quantity of results, an allocation of a linear but age-dependent Young’s modulus to the PDL was carried out.

All structures in the model were assumed to be isotropic.

**Figure 1:**
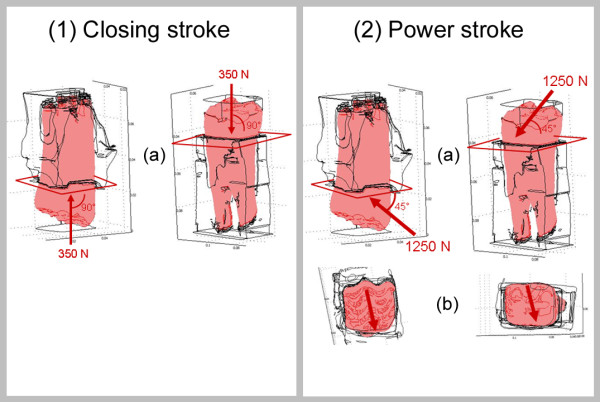
**Force application for closing stroke and power stroke.** Forces were applied perpendicular to a plane through the alveolar crest for closing stroke **(1a)** and at an angle of 45° to this plane for power stroke **(2a)** . Force application occurs along the transverse ridges for power stroke **(b).**

The amount of masticatory forces which were applied in the FE simulations was measured and calculated in previous studies [26,27]. For simulations of the closing stroke a force of 350N [26] was applied perpendicular to a plane described by the alveolar crest (Figure 1). This plane was assumed to be perpendicular to the tooth’s longitudinal axis.

The power stroke was simulated using a force of 1250N. The 3-D orientation of the force vector, applied in FE calculations, was obtained from analyses of the equine chewing cycle [1,28]. According to these data the masticatory forces during the power stroke cause an axial intrusion of the tooth into its alveolar socket both in maxillary and mandibular teeth, but also an important sideward movement (shifting) of the tooth as a consequence of the apparent friction. Due to the movement of the mandible from lateral to medial, maxillary teeth undergo shifting into a palatal direction while mandibular teeth undergo shifting in a buccal direction. This phenomenon was simulated in FE calculations by applying the force at an angle of 45° to the plane through the alveolar crest. In maxillary teeth this force was orientated in a palatal direction while in mandibular teeth it was orientated in a buccal direction (Figure 1). Efforts were made to ensure that the force was applied to the tooth along the transverse ridges of the occlusal surface, which determine the pathway of the mandible during occlusal contact of opposing teeth [1].

A fixation of the outer buccal and palatal/lingual surfaces of the compact bone was chosen as a necessary physical requirement in all calculations.

## Results

### Depiction of finite element simulations

Stresses, occurring in the periodontal ligament in occluso-apical direction (s_z_ normal stress in z-direction, i.e. direction of intrusion) were calculated both for closing and power stroke. Stresses occurring in linguo-buccal or bucco-palatal directions (s_x_ normal stress in x-direction, i.e. shifting direction) were additionally computed for power stroke because this direction was also assumed to be placed under considerable force during this phase of the chewing cycle. Stresses in a mesio-distal direction (s_y_ normal stress in y-direction) were not depicted because the amount of these stresses was insignificant compared to the s_x_ and s_z_ stresses, and irrelevant regarding loading during power stroke. Furthermore, analyses of the SED were conducted for closing and power stroke.

**Figure 2:**
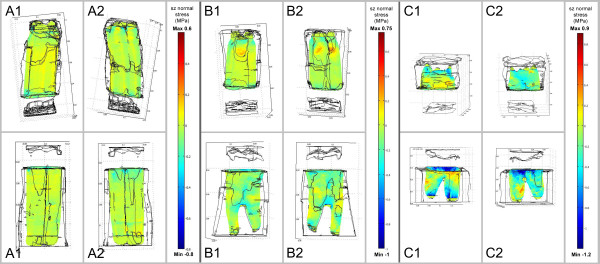
**Stresses occurring in the PDL in occluso-apical direction (sz normal stresses) during closing stroke.** A load of 350N was applied along the tooth’s longitudinal axis. Diagram shows FE results for the three different age groups **(A-C)** on both the shifting side (1) and side where shifting side is averted (2). Tensile stresses are demonstrated by positive scale values (yellow to red), compressive stresses by negative scale values (green to blue). Dimension of stresses increases with increasing tooth age (please note the different age-related scales).

**Figure 3:**
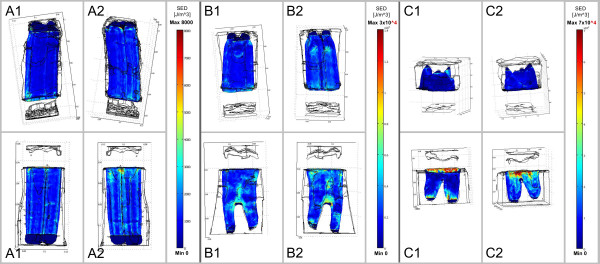
**Strain energy density occurring in the PDL during closing stroke.** A load of 350N was applied along the tooth’s longitudinal axis. Diagram shows FE results for the three different age groups **(A-C)** on both the shifting side (1) and side where shifting side is averted (2). Dimension of SED increases with increasing tooth age (to this please note the different age-related scales).

**Figure 4:**
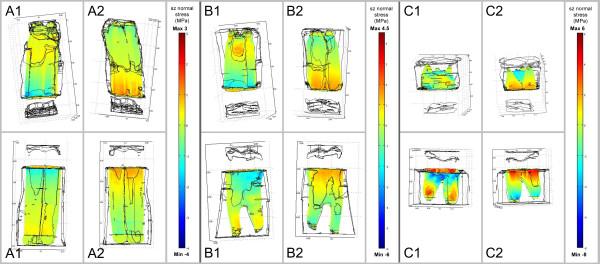
**Stresses occurring in the PDL in occluso-apical direction (s**_**z**_**normal stresses) during power stroke**. A load of 1250N was applied at an angle of 45° to a plane through the alveolar crest. Diagram shows FE results for the three different age-groups **(A-C)** on both the shifting side (1) and side where shifting side is averted (2). Tensile stresses are demonstrated by positive scale values (yellow to red), compressive stresses by negative scale values (green to blue). Dimension of stresses increases with increasing tooth age (please note the different age-related scales).

**Figure 5:**
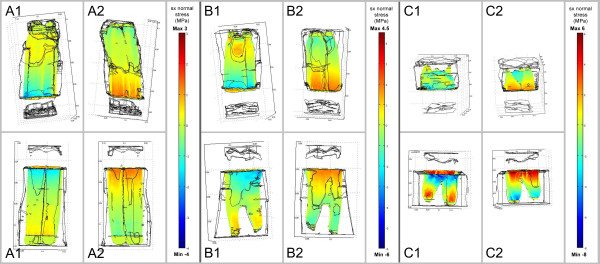
**Stresses occurring in the PDL in bucco-palatal / linguo-buccal direction (s**_**x**_**normal stresses) during power stroke.** A load of 1250N was applied at an angle of 45° to a plane through the alveolar crest. Diagram shows FE results for the three different age-groups **(A-C)** on both the shifting side (1) and side where shifting side is averted (2). Tensile stresses were demonstrated by positive scale values (yellow to red), compressive stresses by negative scale values (green to blue). Dimension of stresses increases with increasing tooth age (please note the different age-related scales).

**Figure 6:**
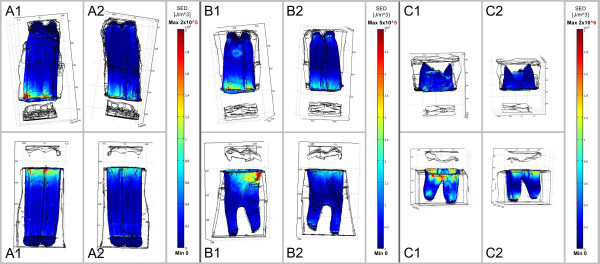
**Strain energy density occurring in the PDL during power stroke.** A load of 1250N was applied at an angle of 45° to a plane through the alveolar crest. Diagram shows FE results for the three different age-groups **(A-C)** on both the shifting side (1) and side where shifting side is averted pushing-averted side (2). Dimension of SED increases with increasing tooth age (to this please note the different age-related scales).

Due to the shifting of teeth during power stroke, for every model a distinction should be made between the side to which the tooth is pressed (the shifting side, i.e. palatal side of maxillary teeth, buccal side of mandibular teeth) and the opposite side (the shifting-opposed side, i.e. buccal side of maxillary and lingual side of mandibular teeth). To be consistent this distinction is used in the depiction of results both for the power stroke and closing stroke (see Figures 2, 3, 4, 5 and 6).

### Finite element simulations

The simulation of the closing stroke showed a uniform distribution of predominantly tensile stresses in an axial direction (s_z_ stresses) within the PDL for the teeth of age group A (young) and B (middle) (Figure 2). In age group C (old) stress distribution appeared irregular, showing tensile but also compressive stresses especially in the mandibular model.

Although the dimensions of these calculated s_z_ stresses during closing stroke increased with tooth age, values were far below those calculated for the power stroke (see below).

The distribution of strain energy densities within the PDL during closing stroke (Figure 3) was quite uniform for all three age groups except for the results of the mandibular tooth of the old age group, where accumulations of SEDs were calculated analogously to the computed stress accumulations in Figure 2. An increase of SEDs with increasing tooth age was also noticed.

During power stroke a specific distribution pattern of compressive and tensile stresses was obtained. In maxillary cheek teeth tensile stresses both in an occluso-apical direction (s_z_ stresses) and in linguo-buccal or bucco-palatal directions (s_x_ stresses) occurred in the PDL in regions near the alveolar crest on the buccal side (shifting-opposed side) and in apical regions of the palatal side (shifting side). In these models with a linear-elastic assumed PDL, compressive stresses (s_z_, s_x_) were calculated in regions near the alveolar crest on the palatal side and in apical regions of the buccal side (Figures 4, 5).

Calculations on mandibular cheek teeth showed similar stress distributions: Tensile stresses occurred near the alveolar crest on the lingual side (shifting-opposed side) and in apical regions of the buccal side (shifting side), while compressive stresses were calculated near the alveolar crest on the buccal and in apical regions on the lingual side (Figures 4, 5). Stresses occurring during power stroke were higher than those occurring during closing stroke.

The highest amplitudes of SED during power stroke occurred in the PDL of both maxillary and mandibular cheek teeth especially in regions near the alveolar crest and also in periapical regions (Figure 6).

Thus, for all age-groups, in both maxillary and mandibular cheek teeth, major stress or SED locations could be observed near the alveolar crest and in periapical regions during power stroke. Furthermore, an increase in stresses and SED with increasing dental age could be noticed.

The fact, that on each side of the tooth (shifting side and shifting-opposed side) both compressive and tensile stresses occurred, showed that the tooth was not uniformly shifted in one direction during the power stroke but that it is rotated in its alveolus around a center of rotation. This could be determined as being approximately in the middle of the reserve crown.

## Discussion

The horse’s masticatory cycle is subdivided into three different phases: opening stroke, closing stroke and power stroke [1,28]. Contact of maxillary and mandibular cheek teeth and thus forces in the PDL occur during the closing and power stroke. Therefore finite element calculations were performed for each of theses phases. During the closing stroke a mean force of 350N [26] occurs in occluso-apical direction along the longitudinal axis of the tooth, which simply causes an intrusion of the tooth into its alveolus. In our calculations this event was represented by a force application perpendicular to the alveolar crest.

The power stroke causes a grinding of the lower jaw teeth upon the upper jaw teeth with a mean load of 1250N [26]. This load is divided into an occluso-apical orientated vector, representing the compression of the maxillary and mandibular teeth against each other, and a linguo-buccal (mandibular teeth) or bucco-palatal (maxillary teeth) orientated vector, describing the shearing action and friction between the teeth due to the sideward movement of the mandible. The ratio of the force of friction between two bodies and the force pressing them together is described by the coefficient of friction (COF).

As no experimental data about the COF during the equine power stroke were available, the assumption of both vectors having the same absolute values (COF = 1) was made to primarily describe primarily the quality of the power stroke. The shifting of the tooth in its alveolus should be simulated but the occurring axial intrusion should not be neglected either. Thus, the force of 1250N was applied to the alveolar crest at an angle of 45°.

In this study, both stresses and strain energy density were calculated. The strain energy density was computed due to the knowledge that this elastic strain energy, stored in the material per volume unit, is one parameter which is related to cell reaction and remodeling processes in bones [29]. Since other studies also describe cell reactions of tendons (for example, in terms of a release of PGE_2_) due to repetitive mechanical loading [30,31] and explain cell movement resulting from strain energy density as a mechanical stimulus [31,32], the strain energy density is also expected to be correlated to tissue responses within the periodontal ligament [33]. The threshold SED, which results in cell reaction, could be variable [29]. Nonetheless, it is expected that the higher the SED the higher the probability of cellular reaction [33,34].

In using the preliminary determined Young’s moduli for the PDL of different age-groups, disregarding the orthotropic behavior of the PDL (different material parameters in different orthogonal directions) must always be taken into consideration when interpreting the calculated stresses and SED. The determined Young’s moduli were actually only precisely determined for dental movement/intrusion along the tooth’s longitudinal axis but not for displacements in other directions (buccolingual/mesiodistal). Because of the age-dependent histology of the periodontal ligament, in terms of changing density and orientation of collagen fibers [35,36], the PDL’s Young’s Modulus is expected to be different for other directions than the axial one. Since stress calculation depends on the Young’s modulus, the absolute amount of stresses occurring in buccopalatal/linguobuccal direction (s_x_ stresses) should be considered with caution.

Further, the allocation of a linear Young’s modulus (instead of a non-linear Young’s modulus) to the PDL with its viscoelastic properties instead represents quite a simplification. It was proved while applying a linear Young’s modulus to the PDL in finite element analyses that calculated compressive forces were higher than those occurring in vivo, whereas tensile forces were definitely underestimated by the model [13]. This is why predictions concerning the absolute quantity of stress dimensions and the occurrence of computed compressive stresses should be interpreted with caution. Nevertheless, such simplifications would not influence the general conclusions of these simulations concerning major localizations of occurring stresses and strain energy density. Thus, it can be expected that the finite element calculations of the closing and power stroke in this study delivered useful and reliable results in view of the understanding of the etiology of periodontal diseases and principles of dental treatment.

During closing stroke the simulations show a quite uniform distribution of low stresses and SED compared to the power stroke. In age-groups A (0–5 years) and B (6-15 years) mainly tensile stresses were calculated, this meeting the expectations of a conduction of chewing forces predominately by tension of collagen fiber bundles [13,23]. Computed compressive stresses in the old age group (>15 years) were calculated because of difficulties concerning an exact force application in axial direction. Due to the anatomical curvatures in cheek teeth, their longitudinal axes are difficult to determine. For this reason, and in terms of reproducibility, the longitudinal axis in these simulations was assumed to be perpendicular to a plane described by the alveolar crest. Such inevitable inaccuracy in axial force application might lead to an inadvertent shifting of the tooth in a distinct direction. Such changes in force application lead to greater displacements in older teeth due to their smaller anchorage surface in the alveolar socket. This explains the computed compressive stresses and SEDs in the mandibular model of the old teeth. Basically, the closing stroke is probably not responsible for the development of periodontal disorders.

The most important phase considering the etiology of periodontal diseases might be the power stroke, where quite high stresses and SED occur in regions near the alveolar crest and around the root tips.

Forces around the alveolar crest might contribute to the development of gingivitis by periodontal damage resulting in gingival pockets, which would deliver a perfect entrance for microorganisms. It is known that periodontal damage in terms of fiber rupture occurs under stresses above 3.8N/mm^2^[37]. In FE calculations this value was exceeded during power stroke especially in the PDL of the old age group. Therefore, on the basis of our results it can be expected that especially old horses are affected by gingivitis, a fact that is already described in literature [5]. The highest stress and SED concentrations in the PDL of the old age group can be explained by the steadily decreasing anchorage surface for the periodontal fibers in aging and shortening teeth. The PDL is the most important tissue for the attenuation and conduction of chewing forces [38]. Thus, it became clear that the exposure of the small PDL of old teeth under chewing forces is higher than that of the large PDL of young teeth.

Further, occurring forces might directly contribute to the development of diastemata which are inevitably found to be associated with periodontal disease [39]. The development of diastemata results from tooth movements. Such movements are proved to be initiated by deformations of the PDL subsequently stimulating the process of alveolar bone remodeling [40]. Thus, it can be expected for the equine dentition that occurring SED in the PDL initiates biomechanical and cellular reactions leading to bone remodeling resulting in diastemata. In our study it was shown that SED in the PDL increases with dental age. This leads to the assumption that the prevalence of periodontal diseases should increase with age, too. This, indeed, coincides with other studies describing periodontal diseases especially in older horses [5,39].

The concentration of forces in apical regions is known to cause local trauma and tissue necrosis [41], which are suitable conditions for the settlement of microorganisms [10]. High concentrations of stresses and SED occurring in the old age group could explain the appearance of periapical infections in old horses. According to our results the described high incidence of periapical infections in young horses [39] cannot be explained by stresses and SED resulting from chewing forces. In young horses the periapical regions are still in an early odontological phase. They are surrounded by a dental organ and dental sack. In these areas proliferative processes inevitably cause massive bone remodeling resulting in the well-known phenomenon of eruption cysts. It is assumed that the osteoclastic action, causing eruption cysts, is initiated by forces within the surrounding tissues. These forces are presumably generated by periodontal tissues but not by masticatory forces [42]. Thus, the hypothesis that high stresses in the periapical region promote the development of anachoresis is supported.

In this study one mean force of 1250N was taken for all calculations in all different age groups to improve reproducibility of single results. However, studies on masticatory forces in horses [26,43] show that forces acting on single teeth vary within a wide range (875N in Triadan position 06 to 1970N in Triadan position 11). The actual forces are influenced by two factors: First, by the position of the teeth, with forces increasing in caudal direction, and second, by the Curve of Spee, which is the curve which connects the cusps of the maxillary teeth and is tangent to the mandibular condyle. Due to the flattening of the Curve of Spee with increasing age, forces acting on single teeth decrease with age [43]. The mean force of 1250N used in our study reflects the forces in Triadan position 09 (for all age groups) quite well. Forces acting in Triadan postions 10 and 11 are known to be higher for all age groups [26]. Thus, in using a mean force of 1250N in our study, calculated stresses and SED might be overestimated, presumably in premolars of old horses, or - more importantly - even underestimated, presumably for Triadan positions 10 and 11.

Remarkably, this assumption is mirrored by the described frequent location of diastemata and following periodontal disorders between second and third molars [44].

FE calculations with defined forces to match individual tooth positions should be performed in future studies to describe the clinical difference of periapical infections subject to Triadan-position.

## Conclusion

Occurring forces in the equine PDL are highest during the power stroke. We propose that high stresses, strains and SED near the alveolar crest and in periapical regions predispose to the development of periodontal diseases and periapical infections by causing local trauma and thus establishing an optimal environment for microbiological settlement. From FE calculations, showing high forces occurring in the PDL of old teeth, a higher prevalence of periodontal diseases in older horses can be concluded.

## Abbreviations

PDL: Periodontal ligament; μCT: Micro-computed tomography; SED: Strain energy density; FE: Finite element; COF: Coefficient of friction.

## Competing interests

None of the authors has any financial or personal relationships which could inappropriately influence or bias the content of this paper.

## Author’s contributions

VC designed the study, performed FE simulations, analyzed data, drafted and wrote the manuscript. ML contributed to the study design, data analysis and interpretation. MG helped performing FE simulations. HS contributed to the study design. CS contributed to the study design, data analysis and interpretation and helped with editing. All authors read and approved the final manuscript.

## References

[B1] Collinson M Food processing and digestibility in horses (Equus caballus) 1994 Doctoral Thesis, Clayton, Monash University

[B2] Frape DL Equine nutrition and feeding 2004 Blackwell Publishing, Oxford

[B3] Baker GJ Some aspects of equine dental disease Equine Vet J 1970 2 105 110 10.1111/j.2042-3306.1970.tb04168.x

[B4] Dixon PM Tremaine WH Pickles K Kuhns L Hawe C McCann J McGorum C Railton DI Brammer S Equine dental disease part 4: a long-term study of 400 cases: apical infections of cheek teeth Equine Vet J 2000 32 182 194 10836472 10.2746/042516400776563581

[B5] Dixon PM du Toit N Dacre IT Easley J, Dixon PM, Schumacher J Equine dental pathology Equine Dentistry 2011 3 Saunders Elsevier, London 129 147

[B6] Crabill MR Schumacher J Pathophysiology of acquired dental diseases of the horse Vet Clin North Am Equine Pract 1998 14 291 307 9742665 10.1016/s0749-0739(17)30199-2

[B7] van den Enden MSD Dixon PM Prevalence of occlusal pulpar exposure in 110 equine cheek teeth with apical infections and idiopathic fractures Vet J 2008 178 364 371 10.1016/j.tvjl.2008.09.026 19010702

[B8] Dacre IT Kempson S Dixon PM Pathological studies of cheek teeth apical infections in the horse: 4. Aetiopathological findings in 41 apically infected mandibular cheek teeth Vet J 2008 178 341 351 10.1016/j.tvjl.2008.09.028 19019712

[B9] Dacre IT Kempson S Dixon PM Pathological studies of cheek teeth apical infections in the horse: 5. Aetiopathological findings in 57 apically infected maxillary cheek teeth and histological and ultrastructural findings Vet J 2008 178 352 363 10.1016/j.tvjl.2008.09.024 19022689

[B10] Bender IB Bender AB Diabetes mellitus and the dental pulp J Endod 2003 29 383 389 10.1097/00004770-200306000-00001 12814220

[B11] Cordes V Gardemin M Lüpke M Seifert H Borchers L Staszyk C Finite element analysis in 3-D models of equine cheek teeth Vet J 2012 10.1016/j.tvjl.2012.02.013 22464401

[B12] Lüpke M Gardemin M Kopke S Seifert H Staszyk C Finite element analysis of the equine periodontal ligament under masticatory loading Wien Tierärzt Mschr - Vet Med Austria 2010 97 101 106

[B13] Cattaneo PM Dalstra M Melsen B The finite element method: a tool to study orthodontic tooth movement J Dent Res 2005 84 428 433 10.1177/154405910508400506 15840778

[B14] Kawarizadeh A Bourauel C Jäger A Experimental and numerical determination of initial tooth mobility and material properties of the periodontal ligament in rat molar specimens Eur J Orthod 2003 25 569 578 10.1093/ejo/25.6.569 14700262

[B15] Ziegler A Keilig L Kawarizadeh A Jäger A Bourauel C Numerical simulation of the biomechanical behaviour of multi-rooted teeth Eur J Orthod 2005 27 333 339 10.1093/ejo/cji020 15961572

[B16] Abé H Hayashi K Sato M Data book on mechanical properties of living cells, tissues, and organs 1996 Springer, Tokyo, Berlin, Heidelberg, New York

[B17] Vollmer D Haase A Bourauel C Semi-automatic generation of finite element meshes of dental preparations Biomed Tech (Berl) 2000 45 62 69 10761287 10.1515/bmte.2000.45.3.62

[B18] Rees JS Jacobsen PH Elastic modulus of the periodontal ligament Biomaterials 1997 18 995 999 10.1016/S0142-9612(97)00021-5 9212195

[B19] Toms SR Eberhardt AW A nonlinear finite element analysis of the periodontal ligament under orthodontic tooth loading Am J Orthod Dentofacial Orthop 2003 123 657 665 10.1016/S0889-5406(03)00164-1 12806346

[B20] Pini M Wiskott HW Scherrer SS Botsis J Belser UC Mechanical characterization of bovine periodontal ligament J Periodontal Res 2002 37 237 244 10.1034/j.1600-0765.2002.00344.x 12200965

[B21] Poppe M Bourauel C Jäger A Determination of the elasticity parameters of the human periodontal ligament and the location of the center of resistance of single-rooted teeth a study of autopsy specimens and their conversion into finite element models J Orofac Orthop 2002 63 358 370 10.1007/s00056-002-0067-8 12297965

[B22] Dorow C Krstin N Sander FG Determination of the mechanical properties of the periodontal ligament in a uniaxial tensional experiment J Orofac Orthop 2003 64 100 107 10.1007/s00056-003-0225-7 12649706

[B23] Pini M Zysset P Botsis J Contro R Tensile and compressive behaviour of the bovine periodontal ligament J Biomech 2004 37 111 119 10.1016/S0021-9290(03)00234-3 14672574

[B24] Picton DC Wills DJ Viscoelastic properties of the periodontal ligament and mucous membrane J Prosthet Dent 1978 40 263 272 10.1016/0022-3913(78)90031-8 100600

[B25] Shibata T Botsis J Bergomi M Mellal A Komatsu K Mechanical behavior of bovine periodontal ligament under tension-compression cyclic displacements Eur J Oral Sci 2006 114 74 82 10.1111/j.1600-0722.2006.00269.x 16460345

[B26] Huthmann S Staszyk C Jacob HG Rohn K Gasse H Biomechanical evaluation of the equine masticatory action: calculation of the masticatory forces occurring on the cheek tooth battery J Biomech 2009 42 67 70 10.1016/j.jbiomech.2008.09.040 19056084

[B27] Staszyk C Lehmann F Bienert A Ludwig K Gasse H Measurement of masticatory forces in the horses Pferdeheilkd 2006 22 12 16

[B28] Bonin SJ Clayton HM Lanovaz JL Johnson TJ Kinematics of the equine temporomandibular joint Am J Vet Res 2006 67 423 428 10.2460/ajvr.67.3.423 16506903

[B29] Huiskes R If bone is the answer, then what is the question? J Anat 2000 197 145 156 10.1046/j.1469-7580.2000.19720145.x 11005707 PMC1468114

[B30] Almekinders LC Banes AJ Ballenger CA Effects of repetitive motion on human fibroblasts Med Sci Sports Exerc 1993 25 603 607 8388071

[B31] Devkota AC Tsuzaki M Almekinders LC Banes AJ Weinhold PS Distributing a fixed amount of cyclic loading to tendon explants over longer periods induces greater cellular and mechanical responses J Orthop Res 2007 25 1078 1086 10.1002/jor.20389 17457818

[B32] Vermolen FJ Gefen A A semi-stochastic cell-based formalism to model the dynamics of migration of cells in colonies Biomech Model Mechanobiol 2012 11 183 195 10.1007/s10237-011-0302-6 21442297

[B33] Carter DR Fyhrie DP Whalen RT Trabecular bone density and loading history: regulation of connective tissue biology by mechanical energy J Biomech 1987 20 785 794 10.1016/0021-9290(87)90058-3 3654678

[B34] Huiskes R Weinans H Grootenboer HJ Dalstra M Fudala B Slooff TJ Adaptive bone-remodeling theory applied to prosthetic-design analysis J Biomech 1987 20 1135 1150 10.1016/0021-9290(87)90030-3 3429459

[B35] Staszyk C Wulff W Jacob HG Gasse H Collagen fiber architecture of the periodontal ligament in equine cheek teeth J Vet Dent 2006 23 143 147 17022193 10.1177/089875640602300303

[B36] Wulff W Histologische Untersuchungen am Ligamentum periodontale des Pferdebackenzahns 2005 Doctoral Thesis, Tierärztliche Hochschule Hannover

[B37] Atkinson HF Ralph WJ In vitro strength of the human periodontal ligament J Dent Res 1977 56 48 52 10.1177/00220345770560011001 264865

[B38] Staszyk C Gasse H Distinct fibro-vascular arrangements in the periodontal ligament of the horse Arch Oral Biol 2005 50 439 447 10.1016/j.archoralbio.2004.10.001 15748697

[B39] Dixon PM Dacre I A review of equine dental disorders Vet J 2005 169 165 187 10.1016/j.tvjl.2004.03.022 15727909

[B40] Bourauel C Freudenreich D Vollmer D Kobe D Drescher D Jäger A Simulation of orthodontic tooth movements – a comparison of numerical models J Orofac Orthop 1999 60 136 151 10.1007/BF01298963 10220981

[B41] Brudvik P Rygh P Non-clast cells start orthodontic root resorption in the periphery of hyalinized zones Eur J Orthod 1993 15 467 480 8112413 10.1093/ejo/15.6.467

[B42] Staszyk C Das Ligamentum periodontale des Pferdebackenzahns: Untersuchungen zur Morphologie unter Berücksichtigung der besonderen Gestalt und Morphodynamik des Pferdebackenzahns 2006 Habilitation, Hannover, Tierärztliche Hochschule

[B43] Huthmann S Staszyk C Jacob HG Rohn K Gasse H Measurement of the Curve of Spee in horses J Vet Dent 2009 26 216 218 20192020 10.1177/089875640902600408

[B44] Dixon PM Acquired Disorders of Equine Teeth Proceedings of Focus on Dentistry 2011 Albuquerque,

